# Using the behavior change wheel to identify barriers to and potential solutions for primary care clinical guideline use in four provinces in South Africa

**DOI:** 10.1186/s12913-018-3778-2

**Published:** 2018-12-14

**Authors:** Tamara Kredo, Sara Cooper, Amber Abrams, Jocelyn Muller, Jimmy Volmink, Salla Atkins

**Affiliations:** 1 Cochrane South Africa, South African Medical Research Council, Cape Town, South Africa; 2 Division of Clinical Pharmacology, Faculty of Medicine and Health Sciences, Stellenbosch University, Cape Town, South Africa; 3 Division of Social & Behavioural Sciences, School of Public Health and Family Medicine, University of Cape Town, Cape Town, South Africa; 4 Dean’s office and Centre for Evidence Based Health Care, Faculty of Medicine and Health Sciences, Stellenbosch University, Cape Town, South Africa; 5 Department of Public Health Sciences, Karolinska Institutet, Tomtebodavägen 18A, 17177 Stockholm, Sweden; 6 New Social Research and Faculty of Social Sciences, University of Tampere, PO Box 100, Tampere, Finland

**Keywords:** Qualitative research, Clinical practice guidelines, Implementation, Primary care, Focus groups, Theoretical domains framework, Behaviour change, Quality improvement

## Abstract

**Background:**

Clinical practice guidelines risk having little impact on healthcare if not effectively implemented. Theory informed, targeted implementation may maximise their impact. Our study explored barriers to and facilitators of guideline implementation and use by South African primary care nurses and allied healthcare workers in four provinces in South Africa. We also proposed interventions to address the issues identified.

**Methods:**

We used qualitative research methods, comprising focus group discussions using semi-structured topic guides. Seven focus group discussions were conducted (48 providers) in four South African provinces (Eastern Cape, Western Cape, Kwazulu-Natal, Limpopo). Participants included mostly nurses, dieticians, dentists, and allied health practitioners, from primary care facilities in rural and peri-urban settings. The analysis proceeded in three phases. Firstly, two analysts conducted inductive thematic content analysis to develop themes of data. This was followed by fitting emergent themes to the Theoretical Domains Framework and finally to the associated Behaviour Change Wheel to identify relevant interventions.

**Results:**

Participants are knowledgeable about guidelines, generally trust their credibility and are receptive and motivated to use them. Guidelines are seen by nurses to provide confidence and reassurance, as well as professional authority and independence where doctors are scarce. Barriers to guideline use include: inadequate systems for printed book distribution, insufficient and substandard photocopies, linguistic inappropriateness (e.g. complicated language, lack of summaries, unavailable in local languages), unsupportive auditing procedures, limited involvement of end-users in guideline development, and patchy training that may not filter back to all providers. Future aspirations identified include: improving the design features of guidelines, accessible places to find guidelines, making digitally-formatted versions available, more supplementary materials (e.g. posters) to support patient engagement, accessible clinical support following training, and in-facility training for all professional cadres to ensure fair access, similar levels of capability and interdisciplinary consistency.

**Conclusions:**

South African primary care nurses and allied health practitioners have high levels of motivation to use guidelines, but face many systemic barriers. We used the Behaviour Change Wheel to suggest relevant, implementable interventions addressing identified barriers. This theory-informed approach may improve clinical guideline implementation and impact healthcare for South Africa.

**Electronic supplementary material:**

The online version of this article (10.1186/s12913-018-3778-2) contains supplementary material, which is available to authorized users.

## Background

Internationally, high-quality, evidence-informed clinical practice guidelines (CPGs) are recognised as essential quality improvement tools [1–3]. CPGs have a range of purposes, intended to standardise care, improve its quality and safety, decrease costs, and improve patient outcomes [3, 4]. They offer a ‘one-stop shop’ for end-users, by providing synthesised information from systematic reviews regarding best practices [5]. However, despite growing availability of CPGs, if not used, they cannot impact on the quality of the care that is delivered.

South Africa has long been developing CPGs, most pronounced during the post-apartheid period when CPGs were considered important tools to redress inequity, standardise care and promote cost-effective care for all. Many CPG development players have been identified: national government, professional societies, hospitals and clinics all contribute according to their needs and agendas [6, 7]. However, despite development and distribution of CPGs, health outcomes remain poor, and generally worse than expected given the per capita health spend relative to other similar middle-income countries [8, 9]. As CPGs aim to optimise care, and yet care appears not to be optimally delivered, it may be helpful to understand the barriers to CPG implementation and use [10, 11].

We know there are no ‘magic bullets’ for improving CPG implementation [12, 13]. Systematic reviews suggest many potential implementation strategies, such as audit and feedback, outreach education and key opinion leaders [14]. Available evidence suggests that tailored, multi-faceted approaches may do better than generic and single-focused interventions [13, 14].

Several pragmatic trials of CPG implementation for lung health, Human Immunodeficiency Virus (HIV) and broader primary care have been conducted in South Africa, finding some improvements when educational outreach is used [15–17]. It is therefore possible that, when used, CPGs may improve health outcomes. If we better understood when and how CPGs are used by South African primary care providers, then CPG developers may design evidence-informed strategies to enhance enablers and overcome barriers.

The Theoretical Domains Framework (TDF) is a useful approach for identifying facilitators of and barriers to behaviour change, and for developing tailored interventions when implementing CPGs [18]. Understanding how best to enhance healthcare providers’ use of CPGs requires consideration of the complex interplay of clinician and patient behaviours, environmental context and social influences. The TDF intends to integrate theories of behaviour change, and bridge health psychology, organisational theory and health services, providing a theoretical basis for implementation research [19]. Several studies have used the TDF to evaluate healthcare implementation challenges or to design theory-informed implementation strategies. Examples include hand hygiene, children’s health checks, human papilloma virus vaccination, dental infections, and lower back pain [10, 11, 20–22]. Some of these explorations further informed the design of complex interventions for research or public health programmes [20].

Utilising the TDF, this study aimed to explore primary care healthcare providers’ perspectives regarding the context, potential barriers to and enablers of CPG use in four provinces in South Africa. Based on the findings, and drawing on concepts from the Behaviour Change Wheel (BCW), this study also sought to provide recommendations for potential interventions to improve CPG usage and implementation.

## Methods

### Theoretical framework

We used a qualitative study design, including semi-structured focus group discussions (FGDs). The overarching conceptual framework used for this article was the Theoretical Domains Framework (TDF) that also formed part of our analysis process described below. The TDF provides a basis to understand behaviours theoretically and therefore target processes most likely to implement desired change [19, 23]. The 14 domains of TDF have been further mapped onto the Capability, Opportunity, Motivation – Behavioural model (COM-B model), a ‘behaviour system’ model which seeks to encapsulate the conditions internal to individuals and those within their social and physical environment necessary for achieving specified behavioural targets [18]. Three essential conditions: Capability, Opportunity, and Motivation (COM-B) are at the core of this system, which posits that these components interact to generate behaviour, which in turn influences them in a back-and-forth cycle. These components form the hub of what is termed a ‘Behaviour Change Wheel’ (BCW), around which are a number of interventions which may be implemented at the individual (e.g. education and training), or policy level (e.g. legislation or fiscal measures) to enable the COM-B elements [18]. The BCW is a practical tool that can be applied in implementation research to move from identifying barriers and enablers to aligning these with tailored interventions [24]. Definitions for the COM-B domains, how they map to the TDF and to the BCW intervention functions are shown (Table 1).

**Table 1:** Links between COM-B, Theoretical Domains Framework and Behaviour Change Wheel intervention functions

COM-B model		Theoretical Domains Framework	Behaviour Change Wheel Intervention functions
Motivation Definition: *all those cognitive processes that direct behaviour, including habitual processes, emotional responding, as well as analytical decision-making.*	Reflective motivation	Professional/ social role and identify	Education, persuasion, modelling
		Beliefs about capabilities	Education, persuasion, enablement
		Optimism	Education, persuasion, modelling, enablement
		Beliefs about consequences	Education, persuasion, modelling
		Intentions	Education, persuasion, incentivisation, coercion, modelling, enablement
	Automatic motivation	Reinforcement	Training, Incentivisation, coercion, Environmental restructuring
		Emotion	Persuasion, incentivisation, coercion, modelling, enablement
Capability Definition: *the individual’s psychological and physical capacity to engage in the activity concerned, and includes having the necessary knowledge and skills.*	Physical capability	Physical skills	Training
	Psychological capability	Knowledge	Education
		Cognitive and interpersonal skills	Training
		Memory, attention and decision processes	Training, environmental restructuring, Enablement
		Behavioral regulation	Education, training, modelling, enablement
Opportunity Definition: *all the factors that lie outside the individual that make the behaviour possible or prompt it.*	Physical opportunity	Environmental context and resources	Training, restriction, environmental, restructuring, enablement
	Social opportunity	Social influences	Restriction, environmental, restructuring, modelling, enablement

### Study settings

South Africa has a population approaching 57 million and a health system invested in primary healthcare [25–29]. The country is currently striving for universal health coverage, publishing a White paper (2015) describing aspects of the National Health Insurance system [30]. Financial federalism is in place in which national government develops strategies, policies and clinical CPGs; and provincial governments implement CPGs, sometimes after adaptation, to healthcare facilities (from regional, to district, to community healthcare facilities) [8].

### Sampling and recruitment

South Africa is a large and diverse country. We therefore selected four of the nine provinces to represent a spectrum of primary healthcare settings: Western Cape, Kwazulu-Natal, Eastern Cape and Limpopo provinces. Each province is different in terms of population size and density, economic development, healthcare spending and resources, and health outcomes (Table 2). While the Western Cape, Eastern Cape and Limpopo have similar population sizes, the Western Cape is better funded, and has higher educational levels, lower levels of poverty and a higher life expectancy than the others. Kwazulu-Natal has the largest population size, a high poverty prevalence and poor life expectancy, despite health expenditure approaching that of the Western Cape. Other factors besides available funds, are likely to play a role in this regard, including high prevalence of infectious diseases, such as HIV [25]. Within each province, we targeted two public sector primary care clinics, one rural and one urban or peri-urban. While we intended to conduct eight FGDs, we completed seven due to delayed access in the Western Cape. To identify clinics, we contacted the provincial research directorates and colleagues working in the provinces for guidance. All healthcare providers working at clinics, regardless of cadre, were invited to participate (Table 3).

**Table 2:** Key health and demographic indicators by South African province^a^

Indicator	Year	Province			
		WC	KZN	EC	LPP
Area as a % of total area of South Africa	2011	10.6	7.7	13.8	10.3
Population	2016	6,279,730	11,065,240	6,996,976	5,799,090
Population % by province	2016	11.3	19.8	12.6	10.4
GDP per capita (USA)	2010	8.69	4.77	3.65	4259
Education level (% population with no schooling)	2015	1.5	6.7	6.1	9.8
Poverty prevalence (food poverty line)	2011	23.2	37.4	40.5	41.5
Population % dependent on public sector	2016	75.96	88.22	90.13	91.58
Health as % of total expenditure	2000	30.0	26.7	20.9	17.8
Per capita public sector health expenditure	2015	4242.5	3623.1	3304.4	2957.7
Life expectancy at birth	2010	68.0	52.9	53.8	63.6
Adult mortality rate (probability of dying between 15 and 60 years)	2010	26.6	52.8	52.2	37.7
Under 5 mortality rate	2015	23.1	57.8	59.6	36.6

^a^*Adapted from South African Health Review 2017* [27]

**Table 3:** Schedule of Focus Groups

Location	Discipline	Number of focus groups (participants)
Western Cape	Nurses, dentists, health promotions officer	1 (*n* = 6)
Eastern Cape	Nurses	2 (*n* = 12)
Limpopo	Dentists, oral hygienist, occupational therapy, physiotherapy, dietician, counsellors, database administrator	2 (*n* = 17)
Kwa-Zulu Natal	Doctors, nurses, quality assurance officer, dentist, physiotherapist, counsellors	2 (n = 12)

### Data collection and management

The FGDs enabled us to explore collective experiences of CPG use at the frontline of healthcare delivery. This method is suited for exploring complexity surrounding CPG use within the context of lived experiences, in ways that encourage participants to engage actively with the research topic [31, 32].

Seven FGDs were held from November 2015 to August 2016. Group sizes ranged from three to eleven participants and lasted from 60 to 90 min. A total of forty-eight providers participated. Primary care providers who took part included nurses, occupational therapists, physiotherapists, dieticians, dentists, oral hygienists and medical doctors.

The FGDs were guided by a semi-structured topic guide which explored the following topics: the context of CPG awareness and use; specific CPGs used (and frequency of usage); access to CPGs; general views and experiences of using specific CPGs; perceptions of barriers to and enablers of CPG use; and recommendations of strategies that might address current barriers to use. The guide was flexible to ensure that participants could express what was important to them, and so learnings from previous FGDs could be clarified and probed further in subsequent FGDs. The FGD guide was not based on the TDF, but rather sought to understand nurses’ perceptions about and experiences with using CPGs on their own terms and their own meaning frames. The TDF was used during the analysis stage to help analyse and organise the data as described below. FGD facilitators received training in facilitation techniques. All FGDs were conducted in pairs; members of the research team (all females) took turns to facilitate.

FGDs were recorded digitally. Reflections and summaries were written after FGDs to capture insights. Initial coding and thematic analysis were conducted after each FGD to guide the sampling process and to ensure data saturation.

FGDs were transcribed verbatim, and transcriptions were reviewed for accuracy by the research team (TK, TM). A few participants including a lay counsellor and entry level nurse chose to share their views using their mother tongue which was not English. A research team member assisted to translate these short sections for us to include in the analysis. Data were stored electronically on password-protected computers; a master list and consent forms were stored in a locked cabinet for which only the project lead had access.

### Analysis

We used an iterative, thematic content analysis approach [31, 33]. Specifically, two researchers read the transcripts (TK, SA) and agreed on the general meaning and central issues presented. One researcher (TK) then re-read transcripts, performing open coding related to general questions posed, including context, use, barriers to and enablers of CPG use, extracting the related quotes [34]. Quotes were then further examined (TK, SA) for manifest and latent meanings [35]. At this point, we searched for conceptual frameworks that might help us better understand and organize the data. The TDF was considered to provide a useful model in this regard, enabling us to encapsulate the individual and context factors that facilitate and /or hinder CPG use that we saw emerging from the data. The model was also deemed valuable to facilitate the subsequent translation of our findings into actionable recommendations for interventions which target specific barriers. This model has been used successfully by others to evaluate healthcare implementation challenges and to design theory-informed implementation strategies [20, 21].

Having examined individual quotes for manifest and latent meanings, two researchers (TK, SC) then used the TDF to further categorise the data. In particular, specific quotations and their meanings were matched to the 14 domains within the TDF. The two researchers performed the matching independently, and subsequently discussed these with each other and the third researcher (SA) to reach agreement and resolve uncertainties. Each quotation was coded to at least one TDF domain, but some we felt could be coded into two or three domains. In the case of the latter, judgments were made about which specific domain the quotes should be categorized, in a manner that captured the meaning of individual quotes and fitted with the broader themes that were emerging. Once our findings were aligned with the TDF domains and associated COM-B system, then proceeded to map the findings onto the respective intervention functions to generate recommendations based on the BCW [24]. The process of developing recommendations was informed by the methods used by Michie and colleagues to link their analysis of the targeted behaviours to appropriate interventions for controlling tobacco and reducing obesity [18].

### Rigour

Credibility was ensured through detailed capturing and description of our approach to sampling, data collection, data management, analysis and interpretation [35]. Consideration of issues regarding reflexivity and transferability were considered throughout the process. Quotations were chosen to provide readers the opportunity to interpret data, establish confirmability and to show the richness of the data. Complementary research competencies and experiences among all researchers influenced data interpretation and strengthened study rigour.

## Results

Most participants were nurses; two were doctors at one FGD in Kwa-Zulu Natal (Table 3). Although we collected limited demographic data, we observed that those in rural facilities had worked for a longer time and lived in the area, whereas at the more urban facilities, participants were generally younger, more recently appointed and potentially more mobile.

In this section we report the potential enablers of and barriers to CPG use in terms of the COM-B domains of ‘Motivation’ (reflective, automatic), ‘Capability’ (psychological, social) and ‘Opportunity’ (social, physical) (Table 1) [23], and reflect on and unpack the TDF categories within them.

### Motivation – Reflective and automatic

Motivation includes behaviors corresponding to reflective motivation and those that are more automatic or habitual. We report on both reflective and automatic motivation as they include issues of emotion, professional identify, beliefs about capability and consequences. Strikingly, across all FGDs, the overwhelming majority of participants expressed motivation to use CPGs. CPG use appeared to evoke a range of positive emotional responses, particularly amongst nurses. Sentiments included *‘reassuring’,* inspiring ‘*confidence’* and providing a sense of autonomy or *‘independence’*. The latter was particularly pronounced in more rural settings, with few doctors:

*It makes [allows] us to be in line with the doctors, it makes us doctors ourself [*sic*], so it means you will be independent* (Nurse_LPP_rural).

Additionally, CPGs were perceived as useful tools to engage the community, share information and protect healthcare providers’ professional integrity, which further motivated use:

*Even if there is a complaint among the community members that we have mismanaged this client, so we say, I have managed this client … through the guidelines and we show him the guidelines* (Nurse_EC_rural).

Overall, CPGs were perceived as credible sources. Nurses and allied healthcare providers in several clinics described having first-hand experience of CPGs improving patient care. One particularly significant example cited was that of HIV, where CPGs had changed rapidly as the field of HIV care changed in South Africa. Providers described having seen patients transition from dying prior to the availability of HIV CPGs, to patients living with HIV after CPGs were implemented. This underscored for them the perceived value that using CPGs bring:

*It’s working, because when we want to find out our statistics, people they are now…[HIV] negative…they have got ARV’s [antiretrovirals] and they are fine…* (Nurse_KZN_rural).

Compared to nursing staff, the link between CPGs, professional identity and enablement seemed lesser for doctors, as one doctor suggested:

*I must confess, we doctors are not very good at seeing this is what the guidelines says. This is the way I do things and then you go on. It’s not just here but if you go to another place you’ll find the same thing.* (Doctor_KZN_peri-urban).

### Capability – Knowledge and skills

Capability includes knowledge, understanding, decision-making and skills as fundamental drivers of behaviour. A consistent narrative amongst participants was that knowledge of CPGs was not a barrier to usage. Participants conveyed considerable awareness of CPGs, with many naming several that were in regular, perhaps even daily use. In addition to knowledge, remembering and deciding to use CPGs was not perceived as a barrier. Some participants even voiced curiosity about why we would conduct research on something that was so obviously part of routine clinical care.

While some participants described using CPGs for ‘*each and every patient’*, others suggested that they were most likely to use CPGs in particular instances. That is, they tended to use CPGs when faced with an unfamiliar clinical case or a change in the recommendations that sparked curiosity, and required learning:

*…what makes me want to read some of them is because I came across such a patient, and I didn’t know what to do then I go back to read. That is what makes me wanna read, otherwise I don’t think I’ll just sit down and read the guideline* (Oralhealth_LPP_peri-urban).

Despite their own knowledge, participants expressed an important gap in CPG awareness amongst patients and the public. Many felt that increasing public awareness of CPGs was important for successful CPG implementation. That is, a more health-literate and empowered public was perceived to encourage accountability of healthcare providers. Several approaches for raising public awareness were proposed, including engaging journalists, use of radio, television and social media:

*Maybe when you’re listening to [the] radio and reading news, they should introduce this change everywhere, because even [the] patients should know* (Oralhealth_LPP_peri-urban).

Another significant gap identified by participants was training in CPG usage. Training was perceived as an essential tool to *‘keep abreast’ or ‘get up to speed’* with CPG content. It was also considered important for enhancing clinical practice and ensuring that all disciplines *‘will be on the same level’* and thus preventing a ‘*clash of information’.* While training was unanimously perceived as necessary for proficient CPG usage, participants were undecided about the setting in which training should take place. Specific feedback about the pros and cons of on-site training and off-site workshops were provided, which are detailed in Additional file 1. Though training was considered key to CPG use, many participants felt that skills building through training was inadequate. Training, regardless of whether providers were from urban or rural settings, was considered insufficient or patchy, not covering all topics and not inclusive of all clinical disciplines. This inadequacy was perceived to result in CPGs which are ‘*hard to interpret’* and thus staff having to *‘struggle’* on their own to use CPGs properly*.* The management process for deciding who would attend workshops was also described as non-transparent and unfair, with ‘*no consistency’* surrounding attendance*.* Thus, while participants were categorical about the need for more training, the issue of how best to do this remains complex.

### Opportunity – Social and physical

Opportunity includes both physical opportunity and social opportunity. Social opportunity considers the social influences that may impact CPG use. While this domain did not generate substantial discussion amongst participants, what emerged consistently, particularly in rural facilities, was the value of supportive social and professional systems as enabling quality clinical care and CPG use. These systems, including involvement of non-governmental organisations, and associated cohesive teams and strong leadership, were perceived to enable the culture of CPG use.

*So it’s team work that matters, if you are working as a team you do* (Nurse_EC_peri-urban).

Whereas we found generally supportive social and professional environments, the physical environment emerged as a considerable obstacle to CPG use. This domain generated extensive discussion, with several sub-themes emerging, namely: the need to adapt to local context; health system challenges; access to CPGs; CPG design needs; and digital CPGs. In addition to describing these barriers in great depth, participants from all disciplines also provided practical recommendations for how these contextual barriers might be addressed.

CPGs being insufficiently adapted to local contexts emerged as a key issue. Given the diversity in a large country like South Africa, the context in which CPGs are used may differ by province. Some CPG recommendations were experienced as *‘not practical’* and not appropriate to local healthcare contexts. Many agreed that for CPGs to become ‘*something that can really apply to us’ and* that *‘actually works to suit the PHC* [primary health care]*’,* healthcare providers should be part of CPG development processes.

Health system challenges emerged as another major barrier to CPG implementation. The ability to operationalise CPG recommendations was described as significantly hindered by *‘no budget’*, *‘slow procurement’*, or the lack of equipment where staff simply *‘don’t have the machine’*. Stock outs of medicines was highlighted as an issue:

*when there is a recommendation and the medication is not there… we are stuck* (Nurse_LPP_rural).

Relatedly, primary care clinic pressures were perceived to limit providers’ ability to properly read CPGs. All cadres described that the *‘long queues outside’* and the time needed to *‘page and page’* through a CPG was not feasible during a consultation.

Participants also identified barriers related to the design, layout and language of CPGs, and made suggestions for how these might be improved to enhance CPG use (Additional file 2). Many spoke about the lengthy nature of CPGs and the *‘big jargon English’,* which limited understanding and use. They expressed a wish for *‘much more user friendly’* CPGs, including using *‘short directive’* and more simple language, and incorporating ‘*summarised’* versions, more definitions, local vernacular and supplementary tools (e.g. posters) to aid understanding and support patient engagement. A doctor suggested that, as people maybe ‘*visual learners’,* use of more attractive and appealing formats, such as graphics, charts, and colour, would enhance CPG use. Colour-coding in one of the primary care CPGs (PC101) was described as effective, as one nurse said, it ‘*keeps you on the toes’* (EC_peri-urban).

Poor access to good quality and up-to-date CPGs materialised as an especially pertinent physical barrier to CPG usage. Many participants, particularly those in rural settings, provided detailed narratives about how *‘hard to reach’* CPGs were. Many described how they frequently *‘get them late’* or have access to ‘*only one copy’* in their clinics. Others spoke about the way in which CPGs are often stored inaccessibly outside of consulting rooms, while others highlighted the poor systems that exist for CPG version control, ultimately resulting in ‘*confusion’* and outdated information. Furthermore, it emerged that even when CPGs are available, they are frequently of sub-standard quality:

*They make copies and pages are missing, the arrangement of the pages, [it] becomes bulkier and all these things. So that’s a problem, I mean people don’t really get the real thing, a reprint or make a copy and make your own.* (Doctor_KZN_peri-urban).

Numerous participants, both rural and urban, highlighted that many of these barriers around access would be addressed if CPGs were available digitally. They explained that access to digital CPGs would enable them to read them in their own time, not only during consultations, which would in turn make keeping up-to-date easier. They also suggested that it would improve knowledge transfer after workshops, reducing issues related to information sharing. Additionally, many believed that digital CPGs would result in all healthcare providers receiving CPGs in a timely manner and further support in-facility capacity building when new CPGs were disseminated.

Despite general agreement that digital CPGs may facilitate usage, a number of complexities associated with this medium emerged. Some participant wondered whether use of digital CPGs in front of patients would generate negative patient perceptions, who might believe that healthcare providers are ‘*busy on Whatsapp’,* accessing other nonwork-related content, or that they lack knowledge. At the same time, while some participants had CPGs on their phones, including the CPG app or electronic books, this was a minority, and mostly seen in peri-urban facilities. Most clinics did not have internet access either via computer stations or wireless internet, and healthcare providers did not consistently have smart phones, data and internet access through other means. This was particularly evident in the more rural clinics where in a FGD of 11 staff, one nurse reported having opened a personal email account, and even that was a recent development. Although participants in the Western Cape FGD described having personal internet access, they suggested that limited phone memory, high data costs and the need to download CPGs at their own expense was a barrier. Thus, use of digital CPGs was described to come with its own set of access issues, and while evidently desirable, remains aspirational from providers perspectives.

### Implications for policy and practice: Theory informed interventions

**Fig. 1:**
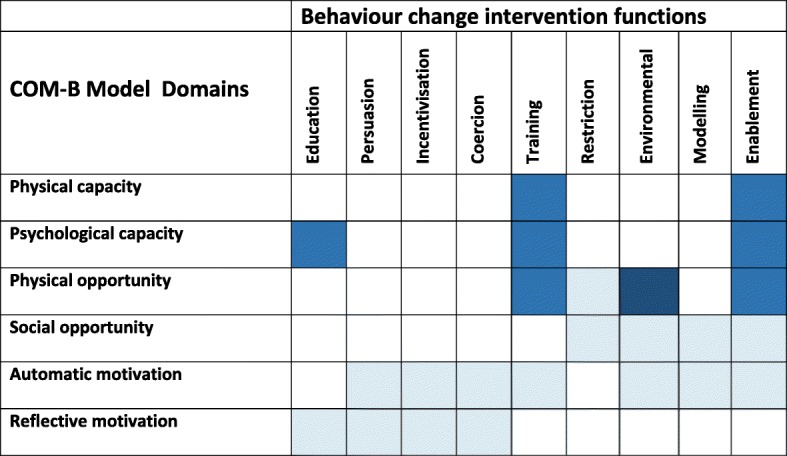
Matrix of COM-B Model barriers and suggested intervention functions. This figure represents a Matrix of barriers that were identified from participants and the potential interventions to overcome them, as guided by the BCW. The matrix is colour coded and all blue coloured areas represent where the COM-B domain aligns with the intervention functions. The darker the shade of blue, the more pertinent the need for an intervention, in light of our findings

The barriers most often expressed by participants were related to the environmental context, resources and training needs. We thus used the BCW approach to map the most relevant intervention functions to address these specific barriers, as shown in Fig. 1 [18]. In this matrix we provide specific suggestions for possible interventions to increase use of South African primary care CPGs.

Therefore, from our findings, ‘physical and psychological capacity’, in particular poorly supported training was a barrier to CPG use; and most strikingly, the ‘physical opportunity’, in that the environmental context and available resources were substantial challenges to CPG use. Based on our results, the following intervention functions are suggested that align the COM-B domain, behavioural barriers and possible interventions:

- Training - imparting skills (for example workshops, on site mentoring and supervision, post-training support)
- Education – increasing knowledge or understanding about specific CPG recommendations (e.g. workshops, post workshop support and clinical support)
- Environmental restructuring – changing the physical environment (e.g. making the CPGs more accessible through different formats, greater design consideration, summarized simple language, more appealing tools that support implementation that help engage patients such as posters and algorithms; ensuring supply chain functioning and access to medicines and equipment; building ICT infrastructure and creating digital access)
- Enablement – increasing means and reducing barriers to increase capability or opportunity (for example, this may include audit and feedback, clinical support and team building).

## Discussion

This study explored the perspectives of primary care healthcare providers, working in public sector clinics, regarding the context, potential barriers to and enablers of CPG use in four diverse provinces in South Africa. We investigated these issues through the lens of the TDF, in order to categorise the barriers and enablers in terms of COM-B: Capability, Opportunity, and Motivation.

### *Enablers – motivation, knowledge and social opportunity.*

Our findings revealed that primary healthcare nurses and allied health practitioners generally trust the credibility of CPGs and are highly motivated to use them. CPG usage was perceived to be associated with a range of positive emotional and professional consequences, experiences that have been described elsewhere as potential benefits of CPGs [3]. In addition, knowledge of CPGs, along with regular use, were reported by most healthcare providers, a finding of importance, given that both knowledge about and positive attitude towards a CPG are factors that have been identified as potentially enhancing CPG implementation [36].

Participants in our study, particularly nursing staff, emphasised the importance of cohesive teams, clinical supervision and strong leadership to enable CPG use. This corroborates reports from higher income settings, which describe the importance of socio-behavioural factors, such as peer support, to enhance CPG use [36–38]. Studies in South Africa have revealed that support and supervision for healthcare providers are currently inadequate. For example, a qualitative study in which allied health practitioners and health managers were interviewed, found a lack of support for allied health practitioners in their practice [39, 40]. Similarly, a recent survey among primary healthcare nurses suggests that many felt unsupported by supervisors to provide best quality clinical care [37, 41]. Against this backdrop, and in light of the findings from our study, enhancing CPG use in South Africa necessitates developing cohesive professional teams and building clinical support for practitioners.

### Barriers – Physical capability (skills and training)

Despite apparent knowledge of CPGs and motivation to use them, patchy and non-inclusive training in CPGs emerged as an important barrier to their usage. Lack of requisite skills and self-efficacy are reported barriers to CPG implementation [36]. The participants in our study considered skills building in CPGs essential for enhancing proficiency to use CPGs properly, ensuring similar levels of capability and knowledge amongst healthcare providers, and for facilitating standardised use across disciplines.

However, exactly how this training should be delivered emerged as a complex issue, with participants suggesting advantages and disadvantages of both on- or off-site training. Our participants talked about off-site educational meetings, on-site educational outreach and supportive clinical audits as desirable. In an overview of systematic reviews, several skills building strategies for implementing health systems in low- and middle-income settings were reported with varying levels of effectiveness, including practice facilitation, educational outreach, audit and feedback, educational meetings, and local opinion leaders [14]. In South Africa, there have been several trials of educational outreach for nurse-driven primary care evaluating CPG implementation [15, 17]. As such, we have supporting evidence regarding feasibility of this approach for managing co-morbidity, and in some studies, evidence of effectiveness for tuberculosis and HIV CPG implementation [15, 17, 42, 43]. Thus, while this study revealed a clear stated need for increased skills building, the best means of providing this in South African primary care might consider using a combination of methods to enable CPG uptake and use.

### Barriers **-** physical opportunity (environmental context and resources)

While other COM-B constructs emerged as enablers of CPG usage, ‘physical opportunity’ materialised as the most substantial barrier, with participants highlighting numerous contextual issues that hinder effective CPG use. These may be further understood as pertaining to two aspects, the CPG itself or the environmental context in which CPGs are implemented.

Regarding the CPG itself, participants perceived that usage of CPGs is hindered significantly when their content is impractical to implement and linguistically inappropriate; when CPG design features are not user-friendly; and if there are inadequate CPG supplementary tools (e.g. pictures) or no summarised versions. This resonates with a review of different features for ensuring CPG ‘implementability’, together with a supporting checklist for CPG developers to consider [44]. These resources suggest that specific features of CPGs are likely to enhance their usage, including structured recommendations; providing contextual information regarding clinical cases; explicit resource implications; and supporting algorithms and clinical tools [44, 45].

In terms of the physical environment, several factors were identified as critical obstacles to CPG implementation. In particular, a lack of necessary equipment and reported budgetary and supply constraints, including stock outs of medicines, were a concern and perceived to be related to poor district or provincial management systems. These health system challenges are well described in the country, including a recent qualitative study in which access to equipment or medicines posed serious challenges to delivery of health services for both users and providers of care [6, 9, 26, 46, 47]. In our study, inadequate systems for distribution of printed CPGs and CPG-related circulars, as well as poor CPG version and quality control, appeared to impact upon CPG use. Taken together, this collection of environmental issues was seen by participants to result in CPGs that are frequently unavailable, inaccessible, of a suboptimal quality and/or difficult to implement. While these barriers emerged across the different study settings, they appeared to be particularly pertinent and heightened in rural areas.

### Aspirational enabler - digital access to CPGs

Participants consistently suggested that making digitally-formatted CPGs and associated technologies (e.g. internet, computers, laptops) available was a key strategy to increase CPG access and use. Digital CPGs were suggested to redress many of the contextual challenges they currently face, such as lack of sufficient CPG hard copies or poor version control. There is growing evidence regarding the role of handheld devices to support CPG use. A systematic review reported that doctors and nurses using a CPG on a handheld device may increase access to information, adherence to a CPG and support for diagnosing conditions, in comparison to peers using paper-based resources [48]. However, despite this promising evidence, results emanate predominantly from high-income settings where access and availability of technologies are different to those in low- and middle-income settings. Therefore, despite interest in this area and fast-growing opportunities in technology, current data costs, lack of infrastructure, internet or devices, particularly in rural settings, present major challenges to this becoming a reality, as revealed in our study.

### Implications for policy and practice: Strategic theory informed interventions to overcome barriers

Given the limited resources to invest in CPG implementation in many settings, ensuring that the interventions best match the issues and barriers that emerge is a rational approach. We identified that investment for implementing primary care CPGs should consider environmental restructuring, enablement, and training and education (Fig. 1).

Training and education is already a major means for delivering information to primary care via regional training centres and responsible district training personnel. However, the results of this and other studies, suggest specific adaptations and enhancements need to be considered and implemented [39, 40] such as enhanced in-facility training and post-training clinical support. Another intervention function is enablement. Given the motivation of healthcare providers to use CPG, further enablement using evidence-based strategies, such as constructive clinical audit and feedback, clinical support and mentoring or team building, may be effective methods to build on the current foundation [17]. Finally, the most substantial barrier, environmental resources, requires considerable resources and planning regarding how best and most cost effectively to restructure the environment to enhance CPG use. Some approaches, such as making more CPG books available or changing the physical appearance of the paper resources enhanced with design features, may be more feasible to achieve short to medium term; however addressing health system reforms including equipment supplies and infrastructure upgrades are important to have on a government agenda for urgent consideration.

Choosing and implementing these interventions will require government buy-in, priority setting and feasibility assessment. Where possible, interventions already in place could be enhanced while others may need to be initiated. The COM-B model is further complemented by a set of specific criteria that can aid decisions when considering interventions. These criteria include: affordability, practicality, effectiveness and cost effectiveness, acceptability, side effects, safety and equity [24]. The relative effectiveness of the priority options should be informed by available systematic reviews [14].

### Limitations

Our study has several limitations. Given the volume of CPGs and CPG users at play in South Africa, we set out with a very broad topic - exploring perceptions of all primary care CPG users for all available primary care CPGs. It is likely we would have identified more specific responses had we evaluated a specific CPG and a specific CPG user. However, given the paucity of published work in our setting, we considered this research exploratory, and the best approach to understanding the state of CPG use in primary care, guiding us to further define the research and policy needs for CPG implementation. A SAGE linked sub-study explored perspectives of allied health workers, adding to our more specific knowledge [39, 40].

Our sample is a fair reflection of the South African public sector primary care, which is predominantly managed by nurses [37]. We have sufficient data on nurse and allied health providers perspectives, however, further work with other cadres is required [40]. The two doctors we spoke to stated that doctors generally do not use CPGs, suggesting that this cadre of professionals may hold differing views to nurses and allied health providers. However, given the small number of doctors participating in our study, it is unclear whether this perspective is widely held by primary care doctors and further exploration is therefore required.

A possible limitation may be the positionality of the researchers in eliciting certain responses [31]. It may be that the presence of researchers asking about CPGs resulted in more positive responses about CPG use, and thus positive reporting bias. However, to pre-empt this possibility, each interview was facilitated by a social scientist, along with a healthcare provider (who understood the clinic context), which we hope brought balance to our interviewing, rather than prompting for specific responses. Given the consistent narratives, regardless of setting, we hope that most participants felt free to provide their true experience and perspective.

We reflected on our choice to use the COM-B and TDF approach, where, following inductive coding, we mapped the codes and themes to the domains of the TDF [18, 23]. We found it assisted us to make sense of the data that emerged, a manner relevant for understanding this aspect of health services research. However, following open coding, the deductive mapping process was challenging. Several of the constructs were related to each other, and could be categorised under more than one domain, for example, professional identity forms a part of social opportunity, and therefore affects motivation. In addition, judgments were required regarding how and where to categorise our findings to best report our understanding of the views of participants. During the process, where items were unclear, we discussed this to resolve discrepancies. In this way, we were able to ensure consistent application of the TDF to our data.

## Conclusions

We found that South African primary care nurses and allied health practitioners are aware of CPGs and have high levels of motivation to use them, however, they face many systemic barriers to doing so. Strategies addressing the most pertinent identified barriers, including physical access to CPGs, training to use them and the equipment and resources to implement CPGs, should build on and enhance processes already in place in South Africa. Prioritising potential interventions, including effective training, clinical audit and feedback, and equipment supply, may strengthen primary care and improve CPG implementation ultimately impacting on the health of South Africans.

## Additional files

Additional file 1: Training: Advantages and disadvantages of training delivered in-facility or off-site. This file reports on perceptions and suggestions of primary care healthcare providers to meet guideline training needs. (DOCX 14 kb)

Additional file 2: Design features for improving use of CPGs. This file reports features that were suggested by participants to improve implementability and use of the available clinical practice guidelines. (DOCX 12 kb)

## Abbreviations

BCW: Behaviour Change Wheel
COM-B: Capability, Opportunity, Motivation - Behaviour
CPG: Clinical practice guideline
EC: Eastern Cape
FGD: Focus group discussion
HIV: Human immunodeficiency virus
KZN: Kwa-Zulu Natal
LPP: Limpopo
PHC: Primary Healthcare
TDF: Theoretical Domains Framework
WC: Western Cape
