# Supplementary material for: Using the behavior change wheel to identify barriers to and potential solutions for primary care clinical guideline use in four provinces in South Africa
Source: BMC Health Serv Res. 2018 Dec 14;18:965. doi: 10.1186/s12913-018-3778-2 (PMC6295099; doi:10.1186/s12913-018-3778-2)
Supplement: Supplementary file 1 — Training: Advantages and disadvantages of training delivered in-facility or off-site. This file reports on perceptions and suggestions of primary care healthcare providers to meet guideline training needs. (DOCX 14 kb) [file 12913_2018_3778_MOESM1_ESM.docx]

**Additional file 1. Training: Advantages and disadvantages of training delivered in-facility or off-site**

|  | **Advantages** | **Disadvantages** |
| --- | --- | --- |
| **Off site** | Builds professional networks (i.e. providers are able to meet/ interact/ share experiences)  *‘good going out for a workshop or training outside because you get to meet the other people from the other facilities and you now come in here to, you know, to see something that is done the same way everywhere else’ WC_d1*  Absence of competing service delivery clinic pressures  Change of environment is enjoyable (e.g. being at hotel, catering) | Non-inclusive/ reduced access for all/ unfair access  *‘District is inviting people to come to these trainings that they are not really, all the stakeholders are not included’ KZN_d1*  Clinician transport expensive and perceived as barrier to attendance  *‘they will have a workshop in Polokwane, and you don’t have transport to go there, or maybe you get the invitation late and so you don’t go there, so they hand out things there and we don’t have it’ LPP_d1*  Inadequate staff back-up at clinics (i.e. lack of human resources makes it hard to send people away)  *‘will have a problem because we have patients here and we can’t attend the meeting all of us’ LPP_d2*  Perceived patchy information sharing/ feedback to other providers (who didn’t attend training)  *‘because if I go alone there sometimes you find that some of the information is so vague that they couldn’t even come here and explain but if you are there as a team then after this everybody knows what is explained there’ LPP_d2*  Challenging and potentially stressful to be away from family commitments  *‘and they take lots of time and you are far from your family’ EC_d2* |
| **On site** | Enhances accessibility i.e. opportunity for more providers to participate and be trained  *‘These things must not be centralized they must go to each and every facility’ EC_d2*  *‘They should come and reach to the rural areas’. LPP_d1*  Teaming-building for colleagues  *‘they come here we are all here as a team they teach us as a team together that knowledge’ LPP_d2*  Facilitates practical case-specific learning | Competing patient pressures/ demands which results in not being able to attend when the workshop is on-site at the facility  *‘to get everybody from the same department at the same time it is, it becomes very impossible in this set up where there’s patients coming’ WC_d1* |
