# Supplementary material for: Using the behavior change wheel to identify barriers to and potential solutions for primary care clinical guideline use in four provinces in South Africa
Source: BMC Health Serv Res. 2018 Dec 14;18:965. doi: 10.1186/s12913-018-3778-2 (PMC6295099; doi:10.1186/s12913-018-3778-2)
Supplement: Supplementary file 2 — Design features for improving use of CPGs. This file reports features that were suggested by participants to improve implementability and use of the available clinical practice guidelines. (DOCX 12 kb) [file 12913_2018_3778_MOESM2_ESM.docx]

**Additional file 2. Design features for improving the use of CPGs**

| **Creating a more accessible hard copy CPG** |
| --- |
| Local and simple language  *So we have to communicate with their language EC_d2*  *I think the guidelines shouldn’t be big jargon English, it needs to be in a phrase form, big sentences big phrases this will confuse us we don’t understand what they are saying. It can be short directive. We are struggling with English sometimes LPP_d2* |
| Summaries and structure  *It is easy to use because there are contents EC_d2*  *something summarised, something much more user friendly LPP_d1*  *if you read EDL on the first page it shows it can even… what is an adherence, what is compliance it should give you definitions so that you can understand what you are talking about. EC_d1* |
| Patient engagement and CPG implementation tools  *she can get a picture just to teach the children and their mothers KZN_d2*  *picture can also be to train the nurses and the doctors, and then you could use it to the client KZN_d2*  *Can show even the pictures if the patient comes with a big wound so we show that on this… and this is the treatment that you are going to get EC­­_d2*  *Is to teach the patient the signs and symptoms of the diseases of what they have… that this is what you have and the patient should know what happens to her……We want more (posters) EC_d2*  *I like posters, if I read sometimes, and I can show the patient marasmus or kwashiorkor, with picture kwashi KZN_d2* |
| Attractive design and format (graphics, charts, colour)  *Baby faces there so they attract you to use them and even the layout EC_d1*  *There are pictures and word pictures EC_d2*  *Even the PC101 is colour coded also so it keeps you on the toes EC_d1*  *EDL. There are no pictures, you know some of us are visual learners and when you see something with your eyes today, tomorrow you can catch it. KZN_d1*  *Posters. More pictures. LPP_d1* |
